# Differential RNA editing landscapes in host cell versus the SARS-CoV-2 genome

**DOI:** 10.1016/j.isci.2023.108031

**Published:** 2023-09-30

**Authors:** Małgorzata Kurkowiak, Sarah Fletcher, Alison Daniels, Paweł Mozolewski, Domenico Alessandro Silvestris, Ewelina Król, Natalia Marek-Trzonkowska, Ted Hupp, Christine Tait-Burkard

**Affiliations:** 1 International Centre for Cancer Vaccine Science, University of Gdańsk, Gdańsk, Poland; 2 The Roslin Institute, Royal (Dick) School of Veterinary Studies, University of Edinburgh, Easter Bush, Midlothian, UK; 3 Infection Medicine, University of Edinburgh, Little France Crescent, UK; 4 Department of Onco-haematology, IRCCS Ospedale Pediatrico Bambino Gesù, Rome, Italy; 5 Department of Recombinant Vaccines, Intercollegiate Faculty of Biotechnology, University of Gdansk and Medical University of Gdansk, Gdansk, Poland; 6 Laboratory of Immunoregulation and Cellular Therapies, Department of Family Medicine Medical University of Gdańsk, Gdańsk, Poland; 7 Cell Signalling Unit, Institute of Genetics and Molecular Medicine, University of Edinburgh, Edinburgh, UK

**Keywords:** Virology, Evolutionary biology

## Abstract

The SARS-CoV-2 pandemic was defined by the emergence of new variants formed through virus mutation originating from random errors not corrected by viral proofreading and/or the host antiviral response introducing mutations into the viral genome. While sequencing information hints at cellular RNA editing pathways playing a role in viral evolution, here, we use an *in vitro* human cell infection model to assess RNA mutation types in two SARS-CoV-2 strains representing the original and the alpha variants. The variants showed both different cellular responses and mutation patterns with alpha showing higher mutation frequency with most substitutions observed being C-U, indicating an important role for apolipoprotein B mRNA editing catalytic polypeptide-like editing. Knockdown of select APOBEC3s through RNAi increased virus production in the original virus, but not in alpha. Overall, these data suggest a deaminase-independent anti-viral function of APOBECs in SARS-CoV-2 while the C-U editing itself might function to enhance genetic diversity enabling evolutionary adaptation.

## Highlights

• RNA-modifying enzymes are upregulated upon SARS-CoV-2 infection *in vitro*
• SARS-CoV-2 VOC Alpha shows a significant increase of C-U RNA edits
• Knockdown of APOBEC3B and 3D increases virus replication of original SARS-CoV-2
• Effect of APOBEC3B and 3D knockdown disappears in VOCs Alpha, Delta, and Omicron

## Graphical abstract

**Figure:**
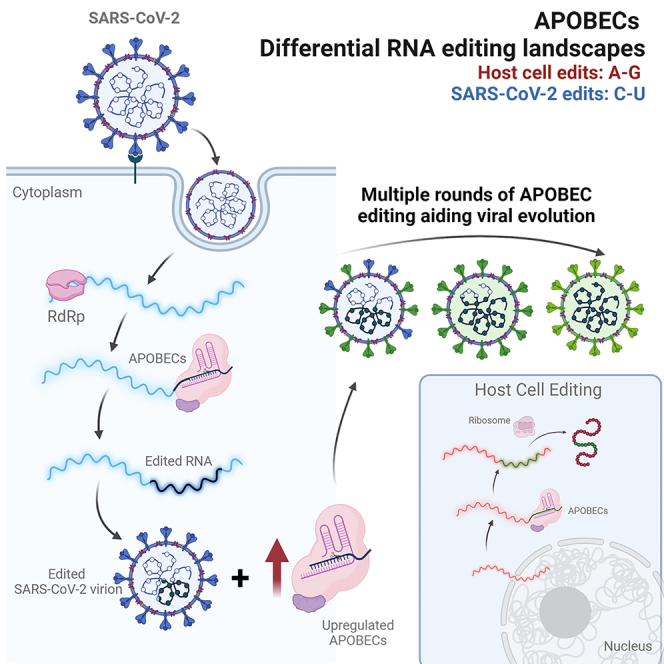

## Introduction

SARS-CoV-2 is a positive-sense RNA ((+)ssRNA) virus with one of the largest genomes of RNA viruses (∼30 kb).^1^^,^^2^^,^^3^ This virus has moderate genetic variability, as it has a proofreading mechanism to correct the errors caused by the RNA-dependent RNA-polymerase (RdRp).^4^^,^^5^ But, according to sequencing data of SARS-CoV-2, constant accumulation of new mutations has been shown, leading to the emergence of new variants and subvariants that can be more virulent, more transmissible, or both.^6^^,^^7^ Two sources of SARS-CoV-2 viral mutations/variants can be distinguished: (i) random errors that are not corrected by the built-in proofreading mechanism and (ii) host antiviral responses that cause viral genome mutations/variants.^8^

Host antiviral responses can introduce mutations/variants in the SARS-CoV-2 RNA genome through three mechanisms: (i) RNA editing mediated by the adenosine deaminase RNA specific (ADAR) enzymes, (ii) RNA editing mediated by the apolipoprotein B mRNA editing catalytic polypeptide-like (APOBEC) family enzymes, and (iii) reactive oxygen species (ROS).^3^^,^^9^^,^^10^^,^^11^ ROS could oxidize nucleic acids to cause viral mutations, hypothesized to be related to the G-U and C-A mutations/variants.^8^^,^^12^

ADAR enzymes edit adenosine (A) to inosine (I) in double-stranded RNA (dsRNA) to cause A-G mutations/variants, which play important roles in immune response and immune regulation. During viral infection, ADARs act directly through hypermutation of the viral RNA (vRNA), or indirectly by editing of host transcripts that modulate the cellular response. ADAR1 regulates numerous sensors detecting intracellular dsRNA, like MDA5, PKR, RIG-I, or OAS, and is essential in triggering response to viral infection. Data on ADAR1-mediated RNA editing of SARS-CoV-2 are contradictory with some studies showing A-G changes,^9^^,^^13^^,^^14^ while others find no clear evidence of ADAR1 editing in SARS-CoV-2.^15^^,^^16^ The analysis by Picardi et al. shows low levels of editing observed early after infection (4 h post infection [hpi]), when ADAR1 and interferon activation is low, while after 24 hpi, higher levels of A-G editing were observed, accounting for <1% of sites.^14^ One also has to remember that sequencing and/or polymerase errors can contribute to the detected substitutions. Moreover, according to the RNA antisense purification and mass spectrometry data, ADAR1 and APOBEC1CF were found as proteins interacting with SARS-CoV-2 RNA.^17^

APOBECs affect the viral genome through C-U hypermutation or through a non-enzymatic mechanism that disturbs reverse transcription. The hypermutation by APOBECs has been widely studied in HIV-1 infections.^18^^,^^19^ However, APOBEC3F and -3G were shown to inhibit HIV replication in non-deaminase dependent manner (without hypermutation).^18^ In influenza A virus (IAV) infection, APOBEC3G and 3F were found to be upregulated during infection; however, overexpression of the proteins did not show an antiviral effect. No assessment of mutation rate was made.^20^ In human coronavirus NL63 (hCoV-NL63), APOBEC3C, F, and H overexpression was found to inhibit virus production but no hypermutation was observed.^21^ ADARs are known to have antiviral function both through regulation of immune function as well as direct editing of RNA viruses through A-I (adenosin to inosine) mutation resulting in A-G and U-C edits. Further details are reviewed in a study by Piontkivska et al.^22^

In SARS-CoV-2 a dominance of C-U substitutions was observed^10^^,^^23^ in up to 52% of observed non-synonymous mutations.^11^ In 18 hospitalized patients, >84% of variations within SARS-CoV-2 accounted for A-G or U-C changes, most at a frequency of <1%.^24^ Due to both the A-G and U-C variation pattern, these were hypothesized to be due to ADAR1 editing, which preferably occurred during replication. As the mutations are not fixed in the infected human population, it has been hypothesized that ADAR1 may play an antiviral role, while RdRp-caused mutations in patients with continued/persistent infection or high viremia are the main source of new SARS-CoV-2 variants.^24^ In cell-based editing, specific SARS-CoV-2 sequences were found to be edited by APOEC1, 3A, and G, while overexpression was not found to have an effect on virus production.^25^ Finally, Kim et al. show experimental evidence that SARS-CoV-2 may take advantage of APOBEC3A-mediated mutational power for SARS-CoV-2 fitness and propagation.^25^

In our study, we applied a pipeline for variant detection and RNA editing analysis based on the REDItools package to detect changes in host as well as the SARS-CoV-2 viral genome using a permissive *in vitro* cell line, Caki-1.^26^ Our data indicate that the number of mutations within the viral genome in EDB-α-1 is higher than in EDB-2, which remains relatively stable throughout passaging. The most frequent substitution type observed in EDB-α-1 strain was C-U, while in EDB-2 no obvious pattern was observed. This indicates that the two strains may use different mechanisms to optimize and maximize the replication efficiency in the infected cells and to avoid the cellular immune response. In contrast to other *in vitro* genome editing experiments (as described previously), we find that, with the original SARS-CoV-2 strain (EDB-2) genome editing may play a much more antiviral role, changing into proviral activity through the emergence of the new variants. Our study adds new data to the current observations in the evolution of the SARS-CoV-2 RNA genome, suggesting that APOBECs play a role in creating new SARS-CoV-2 variants.

While it has been recognized for some time that coronaviruses show a C-U hypermutation rate^11^ possibly linked to an APOBEC-like editing process,^3^^,^^25^ we show here that APOBECs can play a role in RNA editing of SARS-CoV-2 variants. Furthermore, a clear difference in rate of mutation is observed between variants, indicating that adaptation to a host species and recognition by the respective immune system may play an important role in the mutation frequency.

## Results

### ISGs and RNA editing enzymes are upregulated upon infection with SARS-CoV-2 *in vitro*

**Figure 1:**
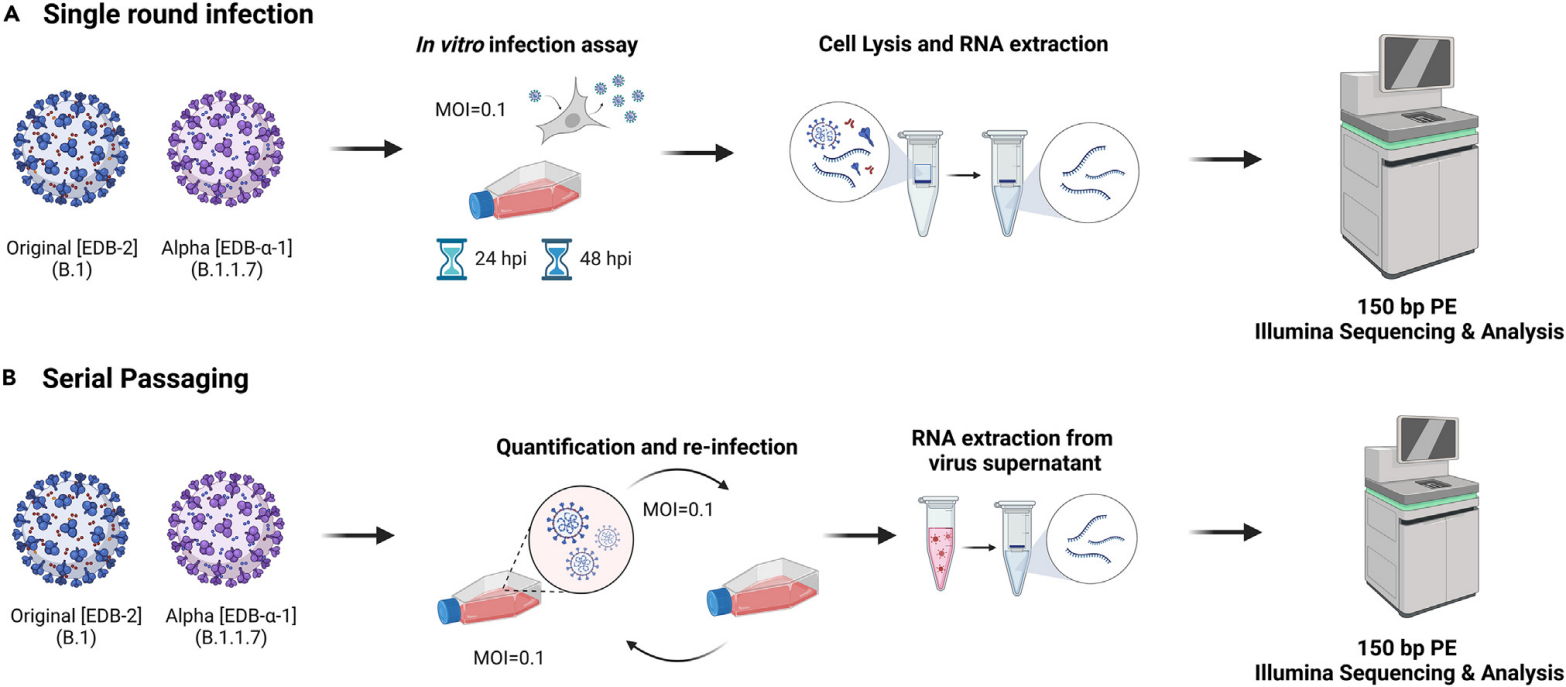
Experimental overview of the methodology performed during this study (A and B) Caki-1 cells were infected using SARS-CoV-2 variants EDB-2 (B.1), or EDB-α-1 (B.1.1.7) at an MOI = 0.1. (A) Cellular RNA was extracted at 24 and 48 hpi or (B) supernatant was harvested at 24 hpi and serially passaged on Caki-1 cells a maximum of four times (P1-4). For each passage of virus, vRNA was extracted from the supernatant. All RNA was amplified prior to Illumina Sequencing. Created using biorender.com.

APOBECs and ADARs are known antivirals and can be stimulated in a virus-specific manner through interferon signaling and RNA-sensing pathways.^27^^,^^28^ For SARS-CoV-2 variants, it is known that they show different sensitivity to different interferons as we (unpublished data) and others have shown.^29^ To investigate the expression of interferon-stimulated, *APOBEC*, and *ADAR* genes, Caki-1 cells were infected with two different patient isolates representing European original (B.1) and the alpha variant of concern (VOC), respectively (strains EDB-2 and EDB-α-1). The Caki-1 cell line, a human kidney-derived cancer cell line, was chosen for being highly susceptible to SARS-CoV-2, other corona- and respiratory viruses, as well as being interferon competent.^26^ Cells were inoculated at MOI = 0.1 with either variant. Following infection, cellular RNAs were harvested at 24 or 48 hpi and subjected to 150 bp paired-end RNA Illumina sequencing analysis (150PE RNAseq; Figure 1A). On average, 95.48% (ranging 95.22–95.74%) of reads mapped to human genome in mock infected (control) cells. At 24 hpi, in cells infected with the EDB-2 strain, 29.95% of reads mapped to human genome and 68.37% of reads mapped to the EDB-2 genome. At 48 hpi, 46.12% of reads mapped to human genome and 51.35% reads mapped to EDB-2 genome. In cells infected with the EDB-α-1 strain at 24 hpi, we obtain 46.10% of reads mapped to human genome and 51.85% of reads mapped to the EDB- α-1 genome. At 48 hpi, 55.60% reads mapped to human genome and 41.74% reads mapped to the EDB-α-1 genome.

To decipher the nucleotide substitution pattern of SARS-CoV-2 variants, EDB-2 and EDB-α-1 were serially passaged on Caki-1 cells up to passage 4 and vRNA was extracted from the supernatant. The vRNA was subjected to 150PE RNAseq analysis and RNA substitutions were detected using the REDItools package (Figure 1B). In each passage, 99.9% of reads mapped to the corresponding EDB-2 or EDB- α-1 genome, respectively. We observed 74 variants with frequency over 1% in EDB-2 P1 (28 variants with frequency over 2%), 57 variants with frequency over 1% in EDB-2 P2 (15 variants with frequency over 2%), 101 variants with frequency over 1% in EDB-2 P3 (30 variants with frequency over 2%), and 73 variants with frequency over 1% in EDB-2 P4 (15 variants with frequency over 2%), while in EDB-α-1 we observed 77 variants with frequency over 1% in P2 (35 variants with frequency over 2%), 135 variants with frequency over 1% in P3 (49 variants with frequency over 2%), and 117 variants with frequency over 1% in P4 (56 variants with frequency over 2%). All variants obtained for examined samples are in Table S1. As already shown in previous studies,^9^^,^^14^ A-to-I candidates as well as C-to-U candidates displayed very low editing levels, less than 1% in more than 99% of positions.

**Figure 2:**
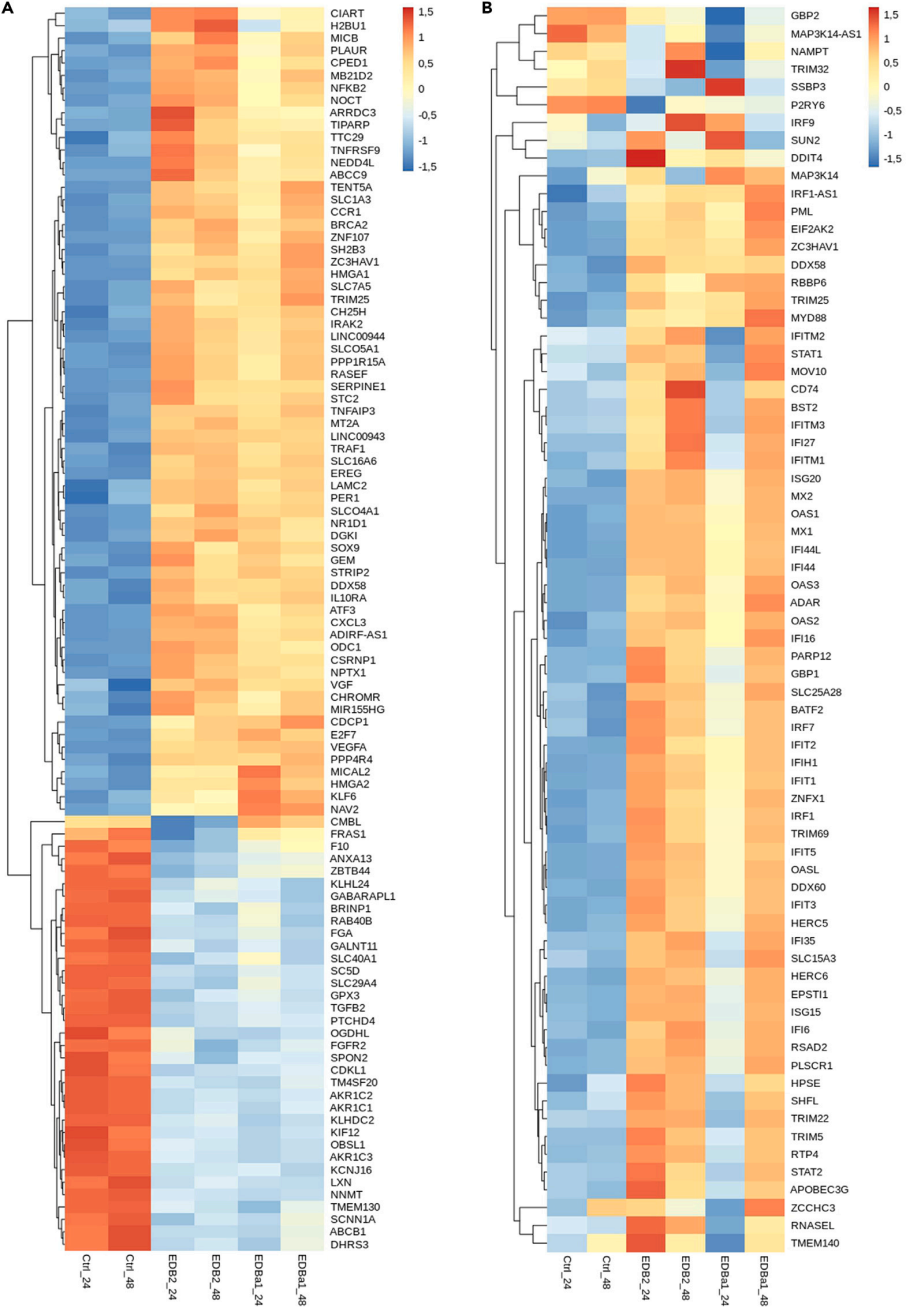
Differential gene expression in SARS-CoV-2 infected cells (A) Top 100 differentially expressed genes in control cells compared to EDB-2 and EDB-α-1 SARS-CoV-2 infected cells The heatmap shows the top 100 differentially expressed genes in mock infected cells (Ctrl) and in cells infected with EDB-2 and EDB-α-1 variants at 24 and 48 hpi. The expression values are normalized and log2 scaled, presented as *Z* score values. (B) A heatmap of selected antiviral interferon-stimulated genes (ISGs) expression in control cells compared to EDB-2 and EDB-α-1 SARS-CoV-2 infected cells. The heatmap shows selected ISG expression levels in mock infected cells (Ctrl) and in cells infected with EDB-2 and EDB-α-1 variants at 24 and 48 hpi. The expression values are normalized and log2 scaled, presented as *Z* score values.

We analyzed whether there are differences in gene expression between control and infected cells to determine whether virus can impact on basal host cell interferon activation, as previously reported.^14^ The heatmap in Figure 2A presents the z-scores of the top 100 differentially expressed genes between different time points post infection in Caki-1 cells. The mock infected cells exhibit visibly different gene expression patterns compared to cells infected with EDB-2 or EDB-α-1 at both 24 and 48 hpi. The average *Z* score at 24 hpi for mock infected cells is −0.38668, rising to 0.257763 for EDB-2 and 0.081738 for EDB-α-1 showing an overall increase in relative gene expression. At 48 hpi with the mock infected control shows an average *Z* score of −0.36137 rising to 0.194584 for EDB-2 and 0.213967 for EDB-α-1. The greatest difference in expression is demonstrated by NNMT (regulator of epithelial-mesenchymal transition^30^), AKR1C2, AKR1C3, AKR1C1 (aldo-keto reductase family members^31^), KCNJ16 (potassium channel^32^), and LXN (inhibitor of zinc-dependent metallocarboxypeptidases^33^). Within the 100 upregulated genes gene ontology (GO) pathways such as import into cell (GO:0098657), response to external stimulus (GO:0009605), and response to stress (GO:0006950) were found to be upregulated with FDRs <0.03 through String DB analysis.^34^ Moreover, from our top 100 differentially expressed genes, three genes (SLC1A3, ZC3HAV1, and DDX58) are also mentioned in the Gencode list of genes involved in the Covid infection with the "updated" status (Gencode: https://www.gencodegenes.org/human/covid19_genes.html, accessed 8/8/2023).

To determine whether the infection with SARS-CoV-2 triggers an immune response cascade in the infected cells related to interferon production and inflammation, we investigated the expression level of interferon-stimulated genes (ISGs), selected on the basis of literature search^35^^,^^36^^,^^37^^,^^38^ (Figure 2B). The relative expression heatmap reveals that cells after 24 and 48 hpi of mock infection present a different expression pattern of ISGs than cells infected with EDB-2 and EDB-α-1 variants. The “classical” ISGs such as MX1, OAS1/2/3 and more recently identified effectors such as APOBEC3G, TRIM5, ISG15, ADAR, IFITM1/2/3, all show a dramatically increased relative RNA expression at 48 hpi for both EDB-2 and EDB-α-1. Of note, the cells infected with EDB-α-1 at 24 hpi exhibit a different pattern of ISGs expression than all the other infected groups (EDB-α-1 at 48 hpi, and EDB-2 at both 24 and 48 hpi). This is also shown in the classical ISGs listed previously, while their relative RNA expression does increase from the control samples it does not reach the over expression phenotype seen in the other time points. This may be linked to the slower replication of EDB-α-1 compared to EDB-2. At 48 hpi, EDB-α-1 gene expression appears more closely related to the EDB-2 48 hpi expression, which could indicate that EDB-α-1 launches a distinct (but overlapping) pathway of anti-viral defense in cells compared to the EDB-2 strain.

**Figure 3:**
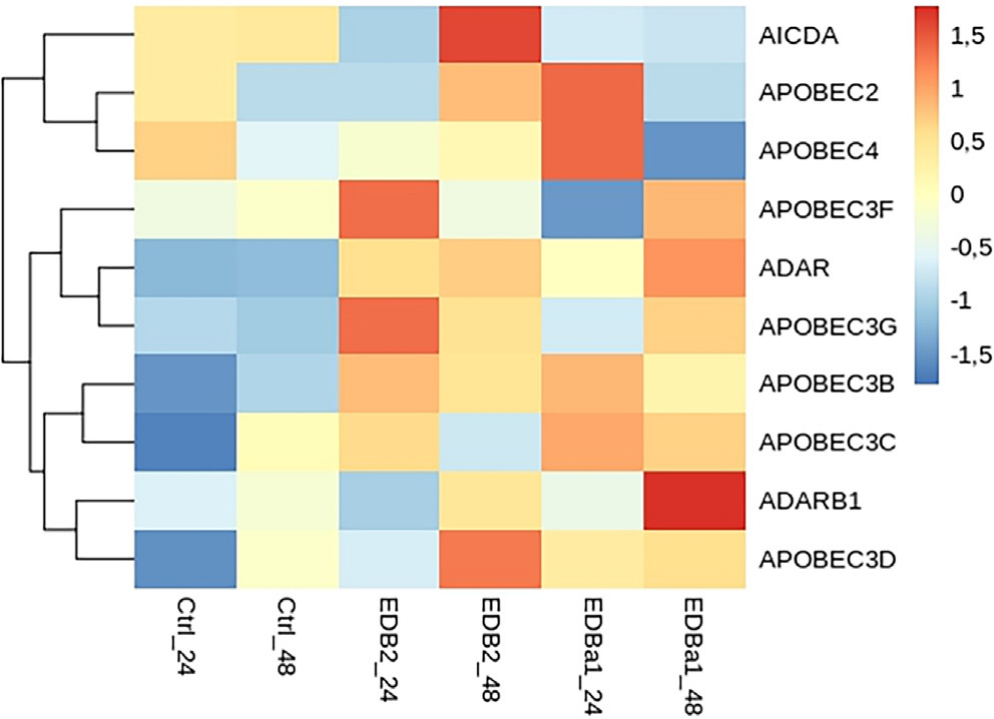
*APOBECs* and *ADARs* gene expression in control cells compared to EDB-2 and EDB-α-1 SARS-CoV-2 infected cells The heatmap presents the expression of *APOBECs* and *ADARs* in mock infected cells (Ctrl) and in cells infected with EDB-2 and EDB-α-1 variants at 24 and 48 hpi. Normalized and log2 scaled, presented as *Z* score values.

ADARs and the APOBEC family also belong to the ISGs. To assess the detailed expression of these RNA editing enzymes, we focused on their expression during infection. These results are presented on the heatmap in Figure 3. Genes related to editing of ssRNA (*APOBEC3B*, -*3G*) and to the editing of dsRNA (*ADAR* and *ADARB1*) show higher expression levels in infected cells compared to mock infected controls. The *Z* score of ADAR’s relative RNA expression increases from −1.218 at 24 hpi in the mock infected, to 0.583 in EDB-2 and -0.022 in EDB-α-1, and increases on these values at 48 hpi (mock infected = −1.183, EDB-2 = 0.717, EDB-α-1 = 1.124). APOBEC3B and -3G, display similar patterns to ADAR. However, *ADARB1* (encoding the ADAR2 enzyme) expression is not consistent throughout infected and mock infected cells, possibly suggesting higher levels of the proteins encoded by the genes mentioned previously and, consequently, elevated activity of the enzymes. As observed for the ISGs as a whole, upregulation of RNA editing enzymes is lower in EDB-α-1 at 24 hpi but largely aligns with EDB-2 by 48 hpi potentially due to the slower replication of EDB-α-1. There are clear exceptions within the EDB-α-1 expression dataset: *AICDA*, *APOBEC2*, and *APOBEC4*, which are downregulated and *ADARB1*, which is strongly upregulated. Overall, the expression of *APOBECs* and *ADARs* is upregulated following infection with both variants of the virus. Therefore, we analyzed the substitutions composition focusing on evidence for RNA editing events.

### C-U substitutions are observed at increased frequency in viral genomic RNA

**Figure 4:**
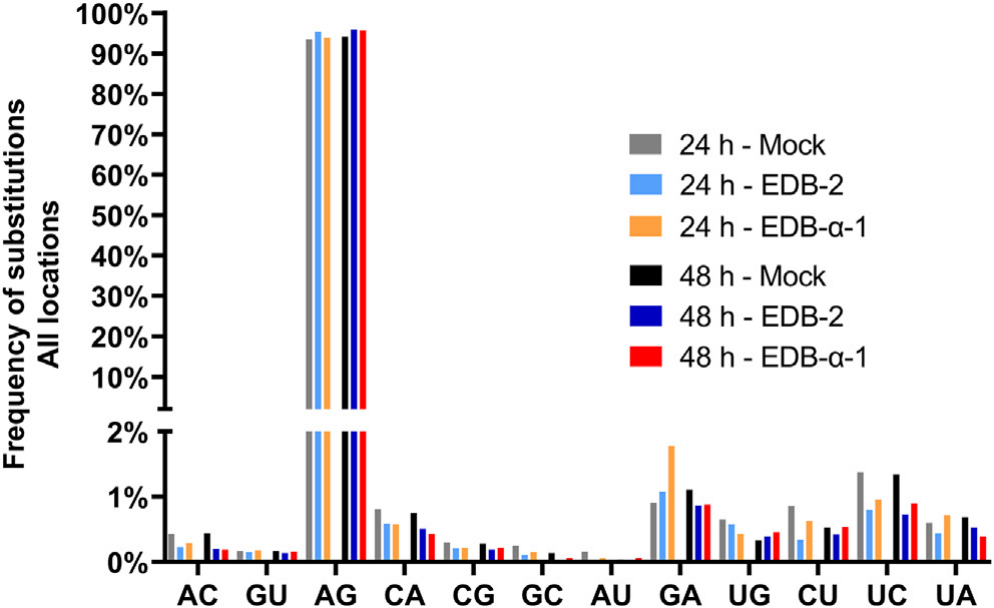
Frequency of host substitutions observed in control cells compared to EDB-2 and EDB-α-1 SARS-CoV-2 infected cells RNA editing events in cellular RNA from mock infected cells (Ctrl) or cells infected with EDB-2 or EDB-α-1 at MOI = 0.1 at 24 and 48 hpi were analyzed using the REDItools package.^56^ The frequency of each type of substitution is depicted as a percentage of the total number of mutations across all locations. The most frequent substitution types observed were A-G, G-A, and U-C respectively.

RNA substitutions in infected cells were profiled at single nucleotide resolution with REDItools applying a stringent filtering scheme (Figure 4) and a list of candidate RNA editing sites was obtained. There were 8,181 sites obtained for 24 h mock control and 84,33 sites for 48 h mock control, while for cells infected with EDB-2 strain, 2,301 sites were obtained after 24 h of infection and 4,214 sites after 48 h of infection. For cells infected with EDB-α-1, 4,000 sites were obtained after 24 h of infection and 5,387 sites after 48 h of infection. All sites obtained for examined samples are in Table S2. By far the most common substitution observed in the host cellular transcriptome (mock and infected with SARS-CoV-2 variants) is A-G, while the second and the third most common substitution types are G-A and U-C, respectively. We observe only a slight increase in the frequency of A-G substitution in infected cells compared to mock infected cells, which may suggest that ADAR1-mediated editing of host RNA is not strongly disturbed by SARS-CoV-2 infection. The slight increase in the frequency of A-G substitution in infected cells is consistent with a predominant activation of ADAR1 by the viral infection. As a control, we analyzed the effects of virus on a transcript of specific host gene, *CDK13*, which is subjected to a relatively high number of A-I edits (ADAR1-specific) in other cancer cell lines (Figure S1).^39^ For example, chromosomal location 39950745 exhibits an increase in A-I edit after EDB-2 or EDB-α-1 at 24 hpi (Figure S1B). On the other hand, location 39950928 has a basal level of A-I edit that is not increased by viral infection hour (Figure S1C). These data highlight that, in Caki-1 cells, regions of the *CDK13* transcript may be impacted by infection with SARS-CoV-2 through over-expression of ADAR1.

**Figure 5:**
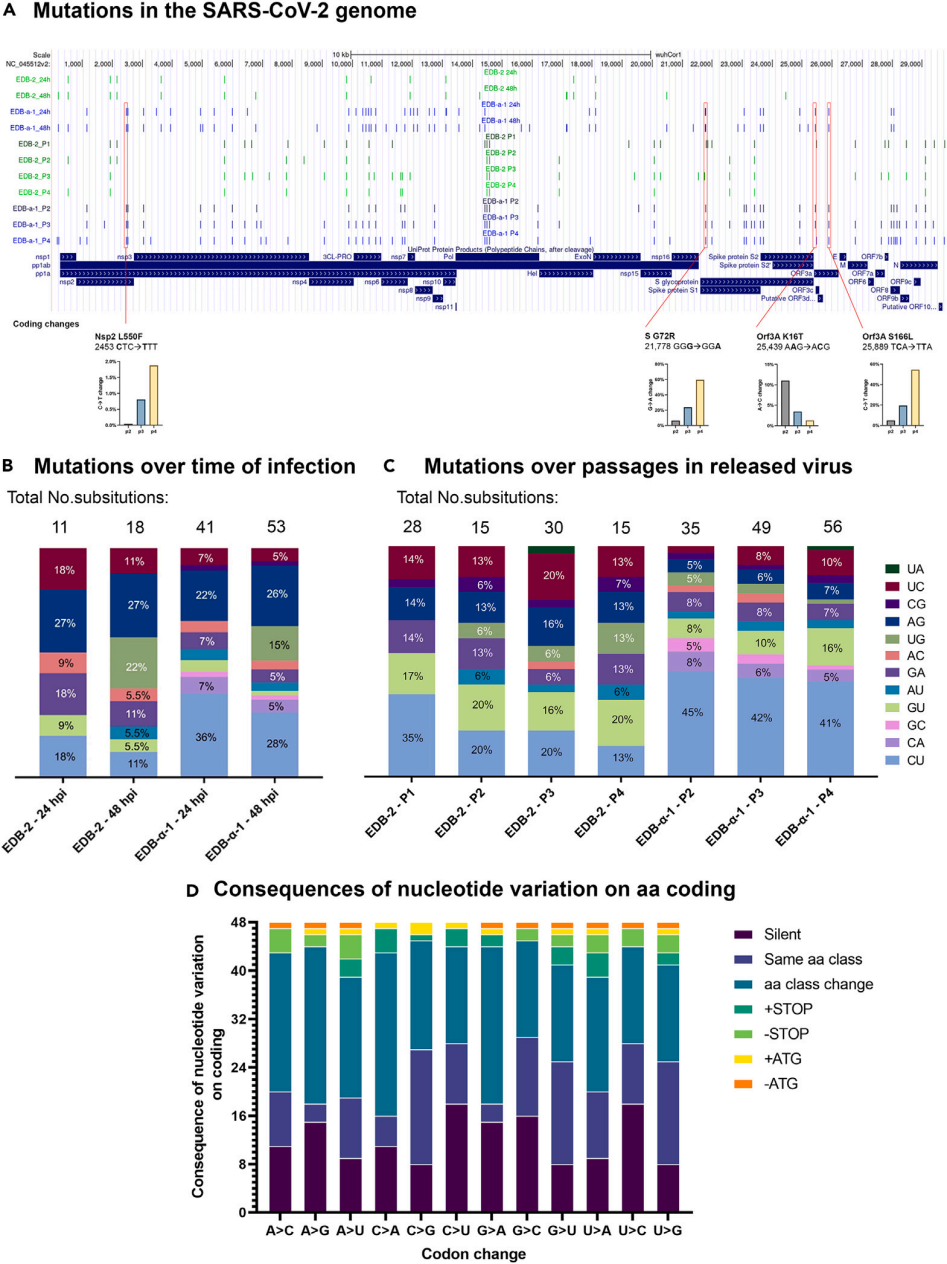
Nucleotide substitutions in SARS-CoV-2 (A) Visualization of identified substitutions location in the SARS-CoV-2 genome from intracellular and culture supernatant-purified RNA. Depiction of the locations of substitutions within the SARS-CoV-2 genome from vRNA amplified and sequenced for EDB-2 (passages 1–4) and EDB-α-1 (passages 2–4) variants during single round infection experiments at 24 and 48 hpi from cellular RNA, or from supernatant over a number of viral passages. Substitutions with the frequency ≥2% were taken under consideration and are presented on the diagram. Highlighted below are the non-silent mutations observed in EDB-α-1 over the course of cell passaging decreasing or increasing in frequency. Absolute numbers may be found in Table S1. (B and C) Frequency of substitutions observed in SARS-CoV-2 variants. Substitutions with frequency of ≥2% were counted. Numbers of substitutions fulfilling those criteria are highlighted above each column. (B) Frequency of substitutions observed in SARS-CoV-2 variants during the time course of infection. Bar colors correspond to the legend in (C). The most frequent substitution observed in EDB-α-1 strain was C-U. The sequences analyzed here were obtained from intracellular RNA. (C) Frequency of substitutions observed in SARS-CoV-2 virus genome as a function of passaging on Caki-1 cells. The most frequent substitution observed in EDB-α-1 strain through passages was C-U. The sequences analyzed here were obtained from the cell supernatant. Significance between the EDB-2 and EDB-α-1 substitutions was calculated using a Student’s *t* test achieving a p value of 0.000183 and a t-value of −5.52954. (D) Consequences on nucleotide variation on amino acid coding. Changes of nucleotides in a codon have been analyzed and categorized as silent (same aa or STOP coding), same amino acid class (non-polar, polar, +charge, -charge), different amino acid class, adding or removing a STOP codon, adding or removing an ATG/Start codon.

We next performed analysis on substitutions located within the genome of EDB-2 and EDB-α-1 variants. Therefore, we focused the REDItools analysis on the vRNA transcripts in the total RNA (rRNA depleted) extracted from infected cells at 24 and 48 hpi. To allow visualization of regions of the SARS-CoV-2 vRNA genome where mutations are potentially more likely to occur, we overlaid substitutions that occurred with a frequency of ≥2% with the Wuhan SARS-CoV-2 sequence NC_045512v2 (Figure 5A). During single-round infection, in EDB-2 we see the same substitutions across both timepoints (24 and 48 hpi), with seven extra and one lost substitutions in the genome at 48 hpi. The same pattern is observed in EDB-α-1, with even distribution throughout the RNA genome across both timepoints (24 and 48 hpi) and 17 extra and 5 lost substitutions in the genome in EDB-α-1 at 48 hpi. The difference here is in the number of substitutions: EDB-α-1 presents significantly more substitutions over time (∗∗∗p value<0.001) that meet the criterium of ≥2% frequency than EDB-2. This indicates that the EDB-2 variant is more genetically stable than EDB-α-1. Moreover, the most frequent substitution observed in EDB-α-1 was C-U while in EDB-2 there was no obvious pattern observed (Figure 5B). These data suggest that there is a distinct (i.e., non-ADAR1) editing of the vRNA or viral genome.

Since the cellular vRNA will be composed of subgenomic transcripts derived from the viral genome as well as some viral genome replicants, we sequenced the viral genome from RNA extracted from the supernatant of viral culture cells, derived from sequential passages. The first passage of EDB-α-1 was not included as not enough material was obtained from the original clinical isolate. Since Caki-1 cells only exhibit CPE at very late time points, the release of subgenomic RNA from lysed cells is small. This was confirmed in the RNA sequencing analysis where cellular RNA reads represented a larger proportion of reads in cell lysate than in supernatant samples.

Similar to the cellular vRNA, EDB-α-1 exhibits an increase in the number of mutations, which amplify over passages (EDB-α-1-P2, 35 substitutions; EDB-α-1-P3, 49 substitutions; EDB-α-1-P4, 56 substitutions with frequency ≥2%). The EDB-2 strain remains consistent in the number of substitutions throughout the passages (Figure 5C), showing no clear trend and overall decreasing numbers of substitutions with passaging (EDB-2-P1, 28 substitutions; EDB-2-P2, 15 substitutions; EDB-2-P3, 30 substitutions; EDB-2-P4, 15 substitutions that meet ≥2% frequency). While no clear prevalent substitution was observed in cellular RNA for EDB-2, the first passage of the supernatant derived virus genomes clearly shows a high rate of C-U substitutions, a pattern observed through passaging for EDB-α-1 (Figure 5C). No clear preference for specific nucleotide substitutions is observed in later passages of EDB-2, this is probably due to the small number of overall mutations throughout the genome. While host RNA appears highly sensitive to the viral activation of ADAR1 and shows high A-G substitutions, the vRNA appears largely unaffected. This is not surprising as the activity of both isoforms of ADAR1 (p110 and p150) can affect the cellular genome, whereas only one of these isoforms can operate outside the nucleus (p150), and therefore have an impact on the cytosolic replication of the virus.^39^ It is also worth noting that in both strains some of the variants that occurred in the first passage are maintained through the next passages. This could hint at the positive role of the RNA editing enzymes in the virus evolution *in vitro* (Figure 5A), but some of these could also be RdRp-mediated substitutions. In Figure 5A we furthermore highlight the four coding nucleotide variations occurring in EDB-α-1 passaging. A C-U variance in the orf1a/orf1ab polyprotein encodes for an L550F (L730F in the orf1a/1ab polyprotein) and increases slowly but steadily over four passages. A mutation in S, G72R, caused by a G-A variance gains prevalence over four passages. Lastly, two coding mutations are found in Orf3A, K16T, and S166L, caused by an A-C and a C-U variance, respectively. While K16T decreases from a low level over passaging, S166L becomes predominant in passage 4.

In Figure 5D we analyzed the potential consequences of nucleotide variation on coding. CU substitutions result in a silent mutation in 37.5% (18/48) variations, the highest level on par with U-C variance. Coding changes retain amino acid class in 10/48 variations. In contrast, A-G variance is silent only in 15/48 variations, and only 3/48 variations retain amino acid class. While no new stop codons are introduced in A-G variation (in contrast to 3/48 for C-U), potentially significant changes to protein function may occur in 62.5% of variations (class change STOP/Start/ATG introduction or removal), whereas this is only the case in 41.7% of variations for C-U.

Together, these data suggest that vRNA within the cell, including the viral genome itself, can be subjected to C-U RNA editing events, which is distinct from the host cell that exhibits largely A-G editing events.

### Knockdown of APOBECs but not ADAR increases viral replication in a variant-dependent manner

**Figure 6:**
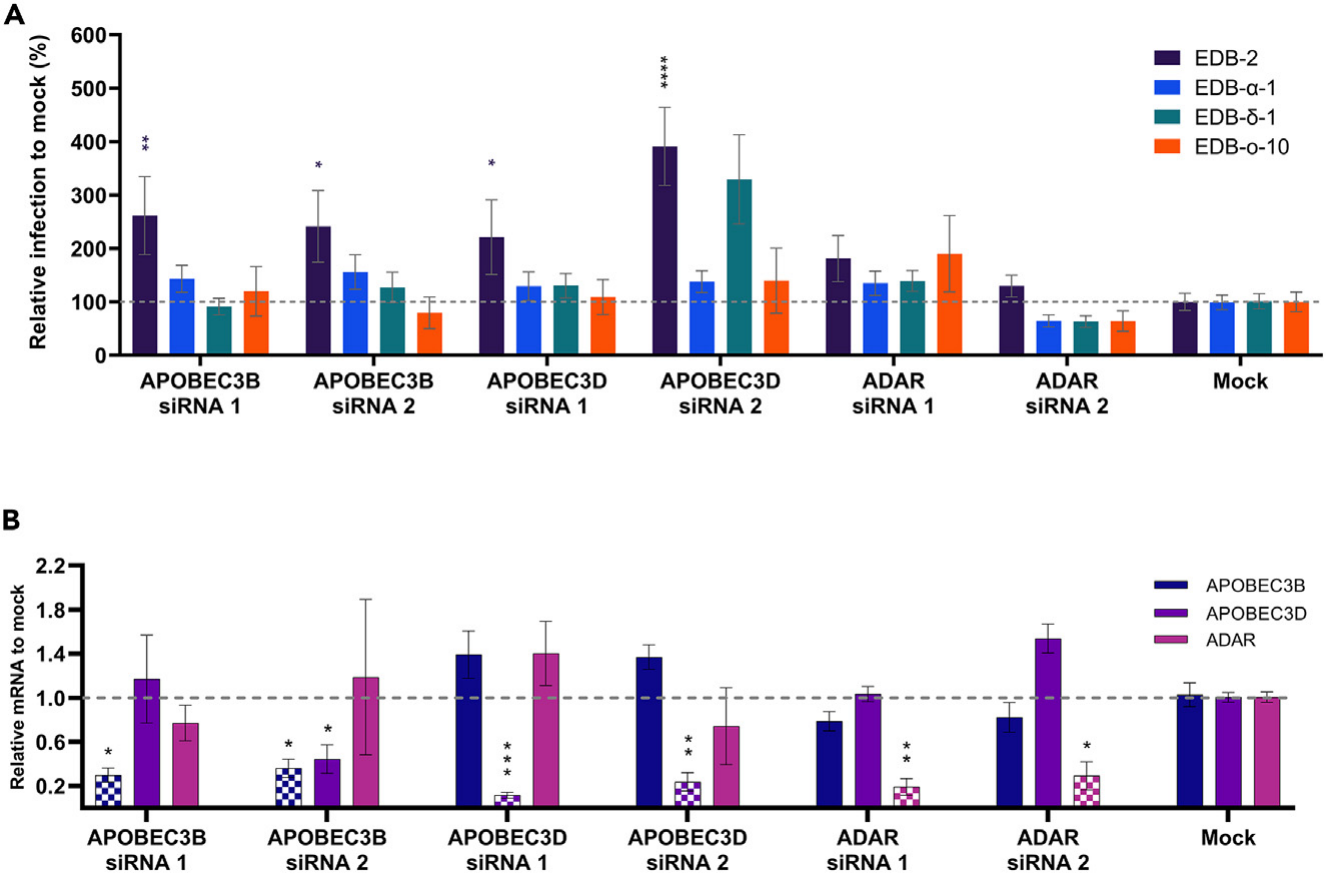
Impact of knockdown of selected *APOBECs* and *ADAR* on virus replication of VOCs on Caki-1 cells Selected *APOBECs* and *ADAR* were knocked down by transfection of Caki-1 cells with RNAi. (A) 72 h post transfection cells were inoculated with MOI = 0.5 of SARS-CoV-2 original, alpha, delta, or omicron VOCs (strains highlighted in the legend). Produced virus was measured at 48 hpi by measuring vRNA in the supernatant through direct lysis RT-qPCR as previously described.^55^ (B) 72 h post transfection RNA was extracted from cells and knock down efficiency was assessed by RT-qPCR. mRNA fold-change was calculated relative to a mock transfected control. Error bars represent +/− the SEM, dashed line indicates no change in replication (100%). Significance was calculated by two-way ANOVA relative to a mock transfected control whereby ∗ denotes p ≤ 0.05, ∗∗ denotes p ≤ 0.01, and ∗∗∗∗ denotes p ≤ 0.0001. N = 3∗3.

Our results thus far suggest that APOBEC-mediated editing (i.e., C-U) is occurring in both EDB-2 and EDB-α-1 viral genomes as observed through the frequency of C-U substitutions. Since vRNA editing through cellular proteins may significantly impact the mutation rate of coronaviruses, we aimed to assess the impact of APOBECs and ADAR1 activity on viral production in different VOCs using RNAi-mediated knockdown in Caki-1 cells. Knocking down gene expression allows for unbiased cell populations and manipulates the endogenous levels of the protein in contrast to protein overexpression. We selected the cytosolic *APOBEC3D* and the nuclear *APOBEC3B* and primarily nuclear *ADAR* (under the caveat that one isoform, ADAR1^p150^, can operate in the cytosol as well as the nucleus^40^). Unfortunately, pre-designed and validated siRNAs for 3F and 3G had previously been deemed non-specific, wherefore were not included in this analysis. Two different pre-designed siRNAs for each of the selected *APOBEC*s and *ADAR* were reverse transfected into Caki-1 cells. At 72 h post transfection, cells were inoculated at MOI = 0.5 with EDB-2 (original), EDB-α-1 (alpha), EDB-δ-1 (delta), or EDB-ο-10 (omicron, BA.1). Virus production was measured by direct lysis RT-qPCR at 48 hpi and visualized as a percentage relative to a mock transfected control (Figure 6A). The knockdown efficiency of the siRNA used to against APOBEC3B, APOBEC3D, and ADAR was assessed for a reduction in the mRNA of all three proteins to account for any non-specific effects (Figure 6B). Knockdown of APOBEC3B with siRNA 2 led to a significant reduction in the mRNA of APOBEC3D in addition to the reduction in the target mRNA of APOBEC3B. All other siRNA resulted in a significant reduction in mRNA for only the target gene.

Knockdown of all *APOBECs*, independent of predominant cellular expression localization, showed a significant increase in production of EDB-2. This suggests that the C-U RNA editing machinery is not playing a positive role in viral propagation, but rather a negative regulatory role. However, despite higher mutation rates, none of the APOBEC knockdowns led to an increased production of EDB-α-1. No increase in virus production could consistently be observed for any of the other VOCs for any of the *APOBECs* across both siRNAs tested. *ADAR* knockdown had no effect on any of the viruses. These data would indicate that, although APOBEC family members are likely driving the C-U RNA editing events in EDB-α-1 as evidence by the ∼40% C-U mutation detection in passages 2–4 of the virions (Figure 5C), there is no correlation between these high levels of C-U editing and virus titer (Figure 6A). Thus, APOBECs are unlikely therapeutic targets, although it is possible that manipulation of the C-U mutation rate could impact on evolutionary fitness.

## Discussion

Gene expression patterns of the top 100 expressed genes in Caki-1 cells change in a similar fashion for both EDB-2 and EDB-α-1 infection. However, at 24 hpi, upregulated genes in EDB-α-1 infections appear less strongly induced. A similar pattern is visible when focusing on ISGs. EDB-2 appears to induce an early and strong induction at 24 hpi, which appears to slightly decrease at 48 hpi. In contrast, EDB-α-1 does not induce as strong an upregulation of ISGs at 24 hpi, but similar overall levels are observed at 48 hpi. The underlying cause could be an adaptation of EDB-α-1 to the human host, improving mechanisms by which to avoid host recognition and stimulation of an interferon response.^41^ Alternatively, early levels of EDB-α-1 production are lagging behind EDB-2 at 24 hpi and response induction may be delayed.^26^ However, EDB-α-1 48 hpi stimulation levels are still below ISG expression levels observed in EDB-2 at 24 hpi.

Among the upregulated ISGs are also nucleotide-modifying enzymes, including ADAR1 and members of the APOBEC3 family. Particularly *APOBEC3B*, *C*, *D*, *F*, and *G* are upregulated in their expression. Previous investigations show that *APOBEC*s appear to be upregulated in a pathogen-specific pattern as shown for hCoV-NL63 and IAV in human airway epithelium cultures.^21^ Whereas hCoV-NL63 was shown to upregulate expression of *APOBEC3A*, *C*, *D*, and *F*, and IAV only upregulated *G* and *H,* it has been shown that *C*, *F*, and *H* possess antiviral activity. Here, we see a clear difference between EDB-2 and EDB-α-1 infection-mediated *APOBEC3* upregulation with alpha showing a downregulation of *F* and *G* at 24 hpi before upregulation at 48 hpi, a pattern also observed for EDB-2 in *APOBEC3D*. In contrast, *APOBEC3 F* and *G* are strongly upregulated in EDB-2 at 24 hpi.

*ADAR* expression patterns in both EDB-2 and EDB-α-1 show an upregulation of this ISG following infection. While host RNA then also shows the highest mutation rates of A-G, this pattern appears to be infection-independent as similar frequencies are observed in mock infected samples. A-G variants are not observed at an increased frequency in vRNA. This is not further surprising since ADAR1 is primarily active in the nucleus^42^ while SARS-CoV-2 replication occurs in the cytosol. Although the interferon-induced p150 isoform of ADAR1 can shuttle between the nucleus and the cytoplasm and so could potentially have a minor effect on the vRNA.^39^ None of the other nucleotide changes appear to increase in frequency in the host RNA following infection, indicating that upregulation of both *ADAR* and *APOBEC*s has little impact on host RNA in general but may affect specific transcripts such as *CDK13*.^43^

Similar to previous observations by Kim et al.,^25^ we found a very high C-U mutation rate in SARS-CoV-2. However, this pattern was much more pronounced in EDB-α-1 (intracellularly and in released virus), whereas EDB-2 only showed increased C-U mutation in early passages of released virus. Due to the low mutation rate in later passages, conclusive preferences of hypermutation are difficult to make for EDB-2. These results, however, are in agreement with previously observed patterns of C-U hypermutation.^11^ Previous observation of broadly prevalent C-U hypermutation also corroborates observations made in this study showing a lack of SARS-CoV-2 strain specificity.

We found three coding mutations increasing in frequency over the course of passaging of EDB-α-1. None of them were linked to previously observed cell culture adaptations linked to the furin cleavage motif.^44^ The mutation in S at G72R has been previously reported to occur in the inactivated, adjuvanted Valneva vaccine as well as in field strains;^45^ however, no further investigations on the function of this mutation are reported. Similarly, the S166L mutation, while observed in some isolates, for example of the B.1.609 lineage, there is no information on the functionality of this mutation. However, the fast positive selection of these two mutations *in vitro* indicates an advantage over other variants in *in vitro* culture.

Following the differential ISG expression experiments, ideally all upregulated APOBEC3 variants would have been tested but due to availability of specific siRNAs we had to focus on 3B and D only alongside ADAR1. Both APOBEC3B, and D knockdowns yielded increased virus production in EDB-2 infected Caki-1 cells but no effect was observed in newer variants including alpha, delta, and omicron. The effect of the APOBEC3B knockdown was slightly unexpected since this nuclear APOBEC protein would not be expected to have a direct effect on SARS-CoV-2. However, it has been identified as an antiviral host factor, and as previously highlighted, deaminase function is not the only route APOBECs function through. They are also involved in the general interferon-stimulated antiviral response (^46^, and reviewed in^47^).

The decreased sensitivity of SARS-CoV-2 variants to APOBEC attenuation is in juxtaposition to the increased frequency of C-U mutations observed in EDB-α-1. Previous experiments have shown that overexpression of APOBECs did not appear to have a negative effect on SARS-CoV-2 production. In contrast, overexpression of APOBEC3 in selected overexpression cell lines increased replication of an unidentified SARS-CoV-2 isolate.^25^ We cannot align the results from this study with our knockdown findings but selection of overexpressing CaCo-2 cells, a cell line known for heterogeneity, may have led to selection of particularly susceptible cells. Previous findings in other hCoVs however show that overexpression of APOBEC3C, F, and H decreased virus production, particularly in hCoV-NL63.^21^ The hCoV-NL63 nucleocapsid was furthermore found to bind the APOBEC3s and through this interaction impacted the level of virus production. This is similar to previous findings where HIV VLPs containing SARS-CoV or hCoV-229E nucleocapsid (fragments) were found to bind APOBEC3G and package the protein into VLPs.^30^

Overall, our results indicate that APOBECs play a role as antiviral ISGs in early variant infections of SARS-CoV-2. Surprisingly, the antiviral effect of APOBECs disappears with the emergence of VOCs and no effect of APOBEC knockdown is observed anymore for alpha, delta, and omicron variants. Future experiments should assess the mutation rate in new VOCs to assess whether higher adaptation to the human host continues to favor C-U mutation or whether eventually an equilibrium is reached. In hCoV-NL63 it was previously observed that APOBECs showed the ability to bind to the nucleocapsid protein.^21^ It should be further investigated whether this interaction leads to an integration of APOBEC into virions as observed for HIV.^48^ Binding efficiency to the SARS-CoV-2 nucleocapsid protein of APOBECs may change with new variants which may be linked to the reduction in antiviral effect of the APOBECs on new VOCs shown in the experiments presented here.

Interestingly, despite APOBEC3B and D showing an antiviral impact on EDB-2, this impact disappeared in VOCs, while C-U mutation rate increased in the alpha variant. This could be a beneficial strategy of CoVs to increase genetic variability of this generally very stable RNA virus.^49^ As shown in Figure 5D, C-U mutation has the highest frequency of silent mutation as a consequence of a base change in a codon. No stop codons or start codons are removed and only in 3 of the possible 48 variations is a stop codon introduced. At the same time C-U mutation decreases the CpG frequency in a viral genome. RNA sequences enriched for CpG dinucleotides are recognized by the zinc finger antiviral protein (ZAP)^50^ While CpG levels in SARS-CoV-2 are already relatively low, CpG reduction is a commonly observed adaptation to mammalian host species and C-U drift or hypermutation is observed in many coronaviruses, including SARS-CoV-2.^11^^,^^47^^,^^51^^,^^52^^,^^53^ It is likely that, similar to seasonal coronaviruses, this increased mutation rate eventually leads to an increase in the frequency of U in SARS-CoV-2. In turn, overall population diversity will probably decrease, after an initial increase following host adaptation, into a more settled state driven by the lack of targets for APOBEC editing and the coronavirus exoribonuclease activity.^11^^,^^47^^,^^51^^,^^54^

### Limitations of the study

Like any cell-based system to study infections, monitoring the effects of cellular responses and the modification thereof on virus replication brings with it limitations. Here, as a model we have used the clear-cell renal carcinoma-derived, Caucasian, male, human cell line Caki-1. Previously, we could show this cell line to be highly susceptible to be infectable with several respiratory viruses.^55^ However, as a cell line model there is no complex interplay between different cell types, which may be observed in an air-liquid interface respiratory epithelial culture, nor is there any interaction with the systemic and cell-cell interaction response observed in complex organisms. Therefore, we have to be aware that we work in a limited system that allows for initial observations and identification of candidate gene interactions impacting virus replication. However, further testing in more complex systems in the future is advisable.

As SARS-CoV-2 continues to infect humans across the globe, it will be interesting to observe further variants in their interaction with RNA modifying enzymes. Findings in this study will also be further informed by the evolutionary drift observed as SARS-CoV-2 continues to infect humans, evading existing immune responses and fully adapting to the human host. They will show how SARS-CoV-2 evolutionary drift compares to common cold coronaviruses, such as hCoV-229E or hCoV-OC43. Both viruses were of zoonotic origin and have adapted to the human population where they continue circulating. C-T drift observed in those viruses and indicatively observed now in SARS-CoV-2 may give us further insight into how future coronavirus introductions into a human host may be risk assessed and predicted in their evolution.

## STAR★Methods

### Key resources table

**Table:** 

REAGENT or RESOURCE	SOURCE	IDENTIFIER
**Bacterial and virus strains**		

SARS-CoV-2-EDB-2	Patient isolate South East Scotland Scottish 279 Academic Health Sciences Collaboration Human Annotated BioResource (reference no. SR1452)	N/A
SARS-CoV-2-EDB-α-1	Isolated from patient South East Scotland Scottish 279 Academic Health Sciences Collaboration Human Annotated BioResource (reference no. SR1452)	N/A

**Chemicals, peptides, and recombinant proteins**		

Roswell Park Memorial Institute-1640 (RPMI)	Sigma-Aldrich	R5886
Heat-inactivated Fetal Bovine Serum	Gibco	10500064
Ultraglutamine-I	Lonza	BE17-605E/U1
MEM Non-essential Amino Acids	Lonza	BE13-114E
Penicillin-Streptomycin (10,000 U/mL)	Gibco	15140148
Opti-MEM	Gibco	31985070
Lipofectamine RNAiMAX Transfection Reagent	Invitrogen	13778500
Trizma hydrochloride solution (pH 7.5, 1M)	Sigma-Aldrich	T2319
IGEPAL CA-630 for molecular biology	Sigma-Aldrich	I8896
Triton X-100	Sigma-Aldrich	T8787
Tween 20	Sigma-Aldrich	P9416
RNase-free NaCl (5M)	Thermo Fisher Scientific	AM9760G
Nuclease-free water	Qiagen	129114
RNasin Plus RNase Inhibitor	Promega	N2611

**Critical commercial assays**		

GoTaq 1-Step RT-qPCR System	Promega	A6020
QIAamp Viral RNA Mini kit	Qiagen	52906
QIAwave RNA Mini Kit	Qiagen	74536
TruSeq Stranded Total RNA Library prep Globin	Illumina	20020612

**Deposited data**		

Raw data after RNA-Seq are deposited in fastq format in NCBI Sequence Read Archive (SRA) database under bioproject nr PRJNA905696	SRA database	https://www.ncbi.nlm.nih.gov/sra/?term=PRJNA905696 and https://www.ncbi.nlm.nih.gov/bioproject/PRJNA905696 Data may also be found as individual accession numbers: SRR22426578, SRR22426574, SRR22426573, SRR22426572, SRR22426571, SRR22426570, SRR22426569, SRR22426568, SRR22426577, SRR22426575, SRR22426567, SRR22426566, SRR22426576

**Experimental models: Cell lines**		

Caki-1	AMSbio	Discontinued; ATCC ref HTB-46

**Oligonucleotides**		

APOBEC 3B fwd GACCCTTTGGTCCTTCGAC	Life Technologies,^57^	N/A
APOBEC 3B rev GCACAGCCCCAGGAGAAG	Life Technologies,^57^	N/A
APOBEC 3D fwd ACCCAAACGTCAGTCGAATC	Life Technologies,^57^	N/A
APOBEC 3D rev CACATTTCTGCGTGGTTCTC	Life Technologies,^57^	N/A
ADAR1 fwd CTGAGACCAAAAGAAACGCAGA	Life Technologies,^58^	N/A
ADAR1 rev GCCATTGTAATGAACAGGTGGTT	Life Technologies,^58^	N/A
RRN18S fwd AGAAACGGCTACCACATCCA	Life Technologies,^59^	N/A
RRN18S rev CACCAGACTTGCCCTCCA	Life Technologies,^59^	N/A
CDC SARS-CoV-2 N3 fwd GGGAGCCTTGAATACACCAAAA	Life Technologies	N/A
CDC SARS-CoV-2 N3 rev TGTAGCACGATTGCAGCATTG	Life Technologies	N/A

**Software and algorithms**		

GraphPad Prism 9.2.0	GraphPad Software	N/A
MxPro qPCR Software	Stratagene	
Spliced Transcripts Alignment to a Reference v2.7.3a	Dobin et al.^60^	https://github.com/alexdobin/STAR/releases/tag/2.7.3a
fastp	Chen et al.^61^	https://github.com/OpenGene/fastp
FASTQC	Andrews^62^	https://www.bioinformatics.babraham.ac.uk/projects/fastqc/
Burrows-Wheeler Aligner v0.7.17	Li and Durbin^63^	https://github.com/lh3/bwa
Samtools v1.11	Li and Durbin^63^	https://samtools.sourceforge.ne
gencode v38		https://www.gencodegenes.org/human/release_38lift37
EdgeR v3.40.0	Robinson et al.^64^	https://bioconductor.org/packages/release/bioc/html/edgeR.html
REDItoolsDnaRnav13.py	Lo Giudice et al.^56^	https://github.com/BioinfoUNIBA/REDItools

**Other**		

APOBEC3B siRNA 1	Ambion	s18411
APOBEC3B siRNA 2	Ambion	s18412
APOBEC3D siRNA 1	Ambion	s44299
APOBEC3D siRNA 2	Ambion	s195730
ADAR siRNA 1	Ambion	s1008
ADAR siRNA 2	Ambion	s1009

### Resource availability

#### Lead contact

Further information and requests for resources and reagents should be directed and will be fulfilled by the lead contact, Christine Tait-Burkard (christine.burkard@roslin.ed.ac.uk).

#### Materials availability

This study did not generate any new unique reagents.

### Experimental model and study participant details

#### Caki-1 cells

Caki-1 (purchased from AMSBio, ATCC reference HTB-46) are a kidney cell line with epithelial morphology isolated from a 49-year old Caucasian male. They have been reported to have a deletion in *CDKN2* (https://www.cbioportal.org/, Accessed 30/10/2022,^65^^,^^66^ also^26^). Cells were authenticated by AMSBio, and were routinely tested for mycoplasma during culturing.

Caki-1 cells were maintained as monolayer cultures in Rosewell Park Memorial Institute (RPMI, Sigma), supplemented with 10% heat inactivated Fetal Bovine Serum (FBS, (Gibco), 1X Ultraglutamine-I (Lonza), and 1X Non-essential Amino Acids (NEAA, Lonza), at 37°C in 5% CO_2_. During infections, 100 U/ml Penicillin and 100 μg/mL Streptomycin (Invitrogen) was added to the culture media.

### Method details

#### Viruses

SARS-CoV-2 variants utilized in this study are EDB-2 (B1.5 at the time, now B.1), EDB-α-1 (B.1.1.7), EDB-δ-1 (B.1.617.2), and EDB-ο-BA.1-1 (B.1.1.529, BA.1) at passage 2 unless otherwise stated.^26^ Infectivity was quantified by endpoint titration on Vero E6 cells (ATCC CRL-1586) with the exception of EDB-ο-BA.1-1 which was isolated and quantified on Caki-1 cells. Briefly, cells were plated to confluence 1-day prior to infection on 96-well plates.

Viral inoculation was performed by diluting virus to the MOI specified in the individual method in serum-free RPMI containing 1X Ultraglutamine-I (Lonza), and 1X NEAA (Lonza) and 100 U/ml Penicillin/100 mg/mL Streptomycin (Invitrogen) at 37°C in 5% CO_2_.

#### Intracellular RNA isolation

To analyze the effect on viral genomes, Caki-1 cells were seeded to confluence in a 12-well plate one day prior to infection. Cells were inoculated with SARS-CoV-2 EDB-2, or EDB-α-1 in serum-free RPMI at MOI = 0.1 for 1 h. Inoculum was replaced by complete medium. At 24 and 48 hpi, the supernatant was removed and the cellular RNA was extracted using an RNeasy mini kit (Qiagen) according to the manufacturer’s protocol. N = 1.

#### Viral RNA isolation

SARS-CoV-2 EDB-2, or EDB-α-1 was serially passaged on Caki-1 cells for up to 4 passages. For each passage, the cells were seeded to confluence 1 day prior to infection at MOI 1. The supernatant was harvested at 24 hpi. VRNA was extracted from 140 μL of supernatant using a QIAamp vRNA Kit (Qiagen) according to the manufacturer’s protocol. N = 1.

#### Intracellular RNA sequencing

Total RNA (rRNA depleted) isolated from infected Caki-1 cells was sequenced with the help of Macrogen sequencing company.

In order to reduce the amount of ribosomal RNA reads, ribosomal RNA was depleted from total RNA using the TruSeq total RNA Library Prep Kit with RiboZero Human/Mouse/Rat (Illumina) according to manufacturer’s protocol prior to being sequenced with a NovaSeq6000 (Illumina, throughput: 8x10^7^ reads per sample, 150 bp read length, paired end, strand oriented). Sequencing was outsourced to Novogene.

#### Viral RNA sequencing

VRNA isolated from the supernatant of infected Caki-1 cells was sequenced by Novogene using the SARS-CoV-2 Genome Library Preparation (Multiplexed Amplicon) with a NovaSeq6000 (Illumina, 1 G raw data per sample, 150 bp read length, paired end, unstranded).

#### Read mapping to human genome

Raw reads were analyzed for quality (FASTQC) and trimmed (FASTP) including adapter and low-quality reads removal. Options used for FASTP were: base phred quality ≥25, 10% unqualified bases in a read allowed, min. read length of 50, discard low complexity reads and polyX trimming in 3′ ends. 25 is the Phred score threshold used for the filtering in the pre-processing phase with FASTP, for REDItools if used with the default parameters the threshold is 30; in the first case it is calculated on the single read and is basically a conventional value based on current practice in the literature, in the second it is applied by position calculated as the average of all the reads in the multi-alignment. After many attempts to find the optimal setting of the parameters those values were chosen because in combination with other filters they gave the greatest signal to noise ratio in the A-to-I editing detection.

The Spliced Transcripts Alignment to a Reference (STAR) v2.7.3a algorithm was used to align trimmed RNAseq reads to the hg38 human genome downloaded from NCBI (GenBank Accession: GCA_000001405.15). STAR was used with the following parameter settings: --outReadsUnmapped Fastx, --outSAMtype BAM SortedByCoordinate, --outSAMstrandField intronMotif, --outSAMattributes All, --outFilterType BySJout, --outFilterMultimapNmax 1, --alignSJoverhangMin 8, --alignSJDBoverhangMin 1, --outFilterMismatchNmax 999, --outFilterMismatchNoverLmax 0.04, --alignIntronMin 20, --alignIntronMax 1000000, --alignMatesGapMax 1000000, with remaining parameters left as default.

Analysis of reads aligned to the human genome (cellular reads) was performed according to the protocol described by Lo Giudice.^56^ Briefly, the output files obtained using Lo Giudice’s protocol were filtered to obtain only variant positions. Variants with 0 frequency were filtered out. Remaining variant positions were annotated with RepeatMasker and dbSNP annotations (from UCSC [University of California Santa Cruz] genome browser). Next, positions supported by ≥ 10 RNAseq reads, min 3 mismatches and min. 0.1 editing frequency were selected. Then *Alu* sites^67^ (ALU), repetitive-non-*Alu* sites excluding sites within Simple repeats or Low complexity regions (REP NON ALU) and non-repetitive sites (NON REP) were selected. ALU, REP NON ALU and NON REP sites were further annotated with known editing events from REDIportal. The next steps were: extraction of known editing events, further filtering, removing duplicates and extraction of RNAseq reads with reference mismatches. Resulting in filtered ALU, REP NON ALU and NON REP sites allowing examination of the distribution of editing candidates for further investigation regarding the fractions of detected substitutions.

#### Read mapping to SARS-CoV-2 genome

Reads unmapped against the human genome were collected and re-aligned with BWA (v0.7.17) mem onto the SARS-CoV-2 EDB-2 and EDB-α-1 reference genomes using default parameters. Virus reference sequences were confirmed by Nanopore sequencing according to the ARCTIC network protocol (https://artic.network/ncov-2019), amplicon set V3, and validated against the patient isolate sequence available on GISaid.

Samtools (v1.11) was used for indexing bam files. To quantify expression at a gene level we used the --quantMode GeneCounts option in STAR and gencode v38 annotation, followed by EdgeR (v3.40.0). RNA-Seq read counts were normalized using TMM normalization method (calcNormFactors function from EdgeR) and converted to logCPM values using EdgeR’s cpm function.

The REDItoolsDnaRnav13.py script^56^ was used to identify all potential RNA variants in samples through alignment to viral genomes. Briefly, in case of identification of variants in viral genomes, the following options were used: -m 30 (for reads mapped with BWA) or 225 (for reads mapped with STAR) (minimum mapping quality score), -v 1 (min. number of RNAseq reads supporting the variation), -q 30 (min. quality score for WGS and RNAseq reads), -e (exclude multiple hits in RNAseq), -n 0.0 (min. RNA editing frequency to be considered), -u (consider mapping quality set with option -m), -l (remove substitutions in homopolymeric regions), -p (use paired and concordant RNAseq reads only). In case of strand-oriented reads we add options -s 2 (infer strand in case of strand-oriented reads), -g 2 (the strand inference type), -S (strand correction). The viral output files fromREDItoolDnaRnav13.py script were filtered to obtain only positions with variants (removing positions without changes), variants with a frequency greater or equal to 0.01 (1%), min. 10 RNAseq reads, min 3 mismatches.

#### siRNA knockdowns

siRNA and Lipofectamine RNAiMax Transfection Reagent (Invitrogen) were combined in OptiMEM (Gibco) to a concentration of 0.1 pmol/μL and 3% respectively. The transfection complexes were incubated at room temperature for 20 min prior to the addition of Caki-1 cells (20,000 per 96-well) to a final siRNA concentration of 10 nM. At 72 h post transfection, cells were either used to extract cellular RNA or were inoculated at an MOI of 0.5 with SARS-CoV-2-EDB-2, SARS-CoV-2-EDB-α-1, SARS-CoV-2-EDB-δ-2, or SARS-CoV-2-EDB-ο-10 for 1 h.

The Silencer Select pre-designed siRNAs (Ambion) used were as follows: APOBEC3B siRNA 1 – s18411, APOBEC3B siRNA 2 – s18412, APOBEC3D siRNA 1 – s44299, APOBEC3D siRNA 2 – s195730, ADAR siRNA 1 – s1008, ADAR siRNA 2 – s1009.

#### Direct lysis SARS-CoV-2 RT-qPCR

Supernatant from knockdown cells that had been infected with SARS-CoV-2 VoCs was harvested at 48 hpi, lysed, and quantified as previously described by Craig et al.^55^ Briefly, an equal volume of supernatant was mixed with VL buffer (10 mM Tris-HCl pH 7.5, 150 mM NaCl, 2.5% Igepal CA-630, 1:2000 RNasin Plus) and agitated at room temperature for 20 min. The lysate was diluted 1:5 with nuclease-free H_2_O and 4.7 μL was used in 10 μL GoTaq 1-Step RT-qPCR reactions using 5 μL of GoTaq qPCR Master Mix, 0.2 μL of GoTaq RT mix and SARS-CoV-2 CDC N3 primers at a final concentration of 350 nM. The reactions were performed on a Stratagen Mx3005 machine according to manufacturer’s protocol (annealing temperature 60°C). The data were normalized against cells that were transfected in the absence of siRNA. This experiment was performed with 3 biological replicates and 3 technical replicates (N = 3∗3).

#### RT-qPCR of intracellular RNA

Total RNA was extracted from knockdown cells in 12-well plates using the RNeasy Mini kit according to the manufacturer’s instruction. RNA levels of APOBEC3B, APOBEC3D, and ADAR were analyzed by RT-qPCR using the GoTaq 1-Step RT-qPCR kit (Promega) according to the manufacturer’s instructions using primers at a final concentration of 250 nM and 30 ng of RNA. Annealing temperature was set at 60°C and reactions were carried out on an Agilent Mx3005. This experiment was performed with 3 biological replicates and 3 technical replicates (N = 3∗2).

### Quantification and statistical analysis

Details on quantification of genome reads and dataset analysis may be found in the respective experimental sections above.

To compare the impact of APOBECs and ADAR on SARS-CoV-2 infection, two-way ANOVA was performed comparing the mock transfection populations to each siRNA knockdown population. Statistical analysis and graphs were produced using GraphPad prism v9.1.0 on 3 biological replicates and 3 technical replicates (N = 3∗3) in EDB-α-1, EDB-δ-1, and EDB-BA1.1, and 5 biological, 3 technical repeats (N = 5∗3) for the original European strain, EDB-2.

To assess the knockdown efficiency of siRNAs, Dunnett’s multiple comparisons test was used to compare the mock transfected populations to each siRNA knockdown. Statistical analysis and graphs were produced using GraphPad prism v9.1.0 on 3 biological replicates and 2 technical replicates (N = 3∗2).

## Data Availability

• RNA-seq data have been deposited at NCBI Sequence Read Archive (SRA) database under BioProject nr PRJNA905696 (SRA: https://www.ncbi.nlm.nih.gov/sra/?term=PRJNA905696 and BioProject: http://www.ncbi.nlm.nih.gov/bioproject/905696) and are publicly available (released on 2023-07-10). Accession numbers are listed in the key resources table. • This paper does not report original code. • Any additional information required to reanalyze the data reported in this paper is available from the lead contact upon request. • Any raw data used for the analysis of experimental data will be shared on reasonable request to the corresponding authors.

## Supplemental information

Document S1. Figure S1 and Tables S1–S4

Table S1: Variants obtained for EDB-2 and EDB-α-1 strains.

Table S2: Substitutions observed in the host cellular transcriptome (mock and infected with SARS-CoV-2 variants).

Table S3: Gene expression data underlying figures 2 and 3.

Table S4: Bioinformatics pipeline.
