# Supplementary material for: Differential RNA editing landscapes in host cell versus the SARS-CoV-2 genome
Source: iScience. 2023 Sep 30;26(11):108031. doi: 10.1016/j.isci.2023.108031 (PMC10590966; doi:10.1016/j.isci.2023.108031)
Supplement: Document S1. Figure S1 and Tables S1–S4 [file mmc1.pdf]

iScience, Volume *26*

Supplemental information

Differential RNA editing landscapes in host cell versus the SARS-CoV-2 genome

Małgorzata Kurkowiak, Sarah Fletcher, Alison Daniels, Paweł Mozolewski, Domenico Alessandro Silvestris, Ewelina Król, Natalia Marek-Trzonkowska, Ted Hupp, and Christine Tait-Burkard

Chr	Position	Ref	Ed	In dbSNP	Gene	Region	Number of Edited samples	Edited site annotated in public databases
7	39950700	A	G	No	*CDK13*	Exonic	1505	ATLAS, RADAR
7	39950703	A	G	Yes	*CDK13*	Exonic	3284	ATLAS, RADAR
7	39950745	A	G	No	*CDK13*	Exonic	2880	ATLAS, RADAR
7	39950928	A	G	No	*CDK13*	Exonic	1735	ATLAS, RADAR
7	39950949	A	G	No	*CDK13*	Exonic	2927	ATLAS, RADAR
7	39950979	A	G	No	*CDK13*	Exonic	1103	ATLAS, RADAR
7	39950991	A	G	No	*CDK13*	Exonic	1698	ATLAS, RADAR

A

Locations: 39950700 and 39950703:

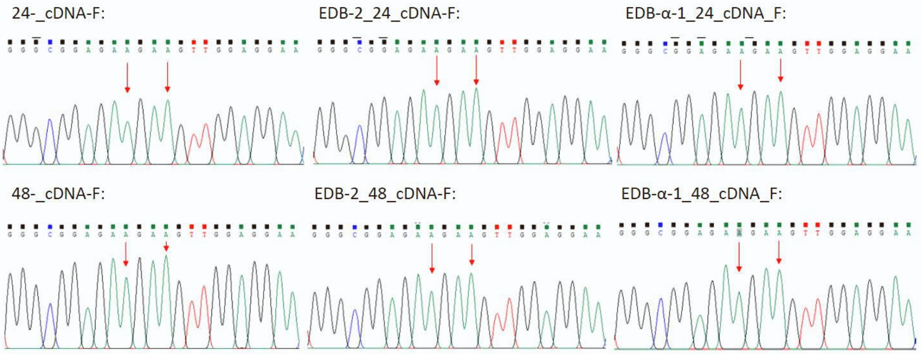

B

Location: 39950745:

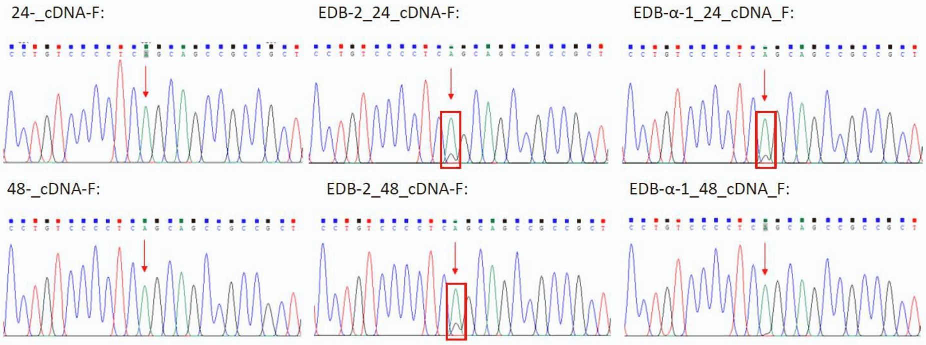

C

Locations: 39950928 and 39950949:

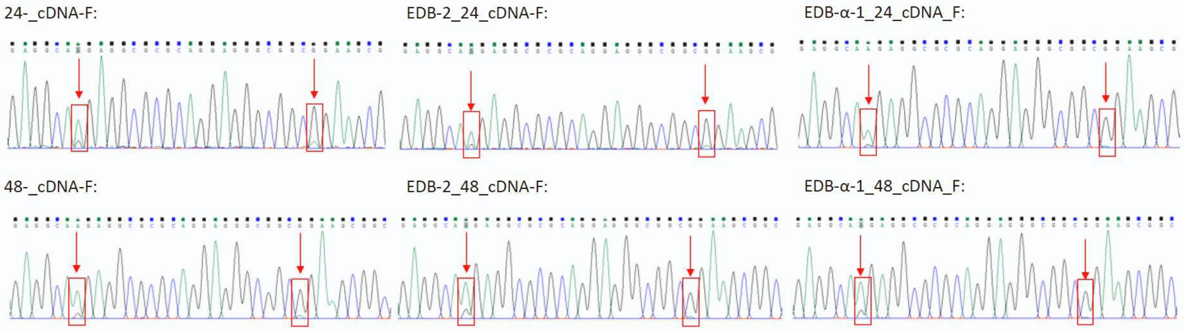

D

Locations: 39950979 and 39950991:

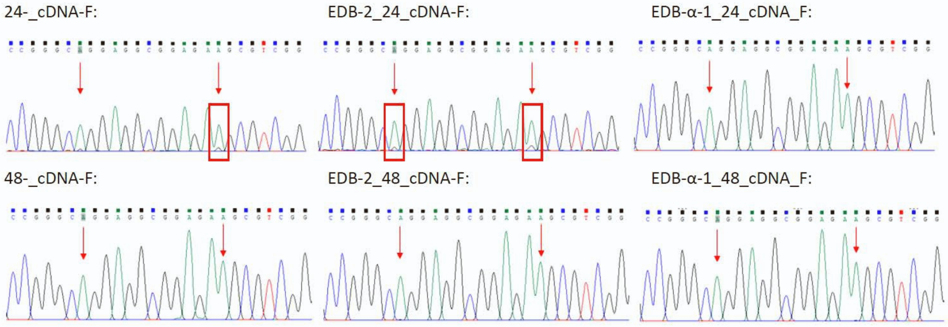

Supplementary files legends and titles:

Supplementary Figure 1. Sanger sequencing results for a ~300bp potential editing region in *CDK13* transcript, related to figure 4. Representation of the edits deposited in REDIportal within a 290 nt region surrounding the analyzed edits in *CDK13* gene (Kurkowiak et al. 2021). (**A-D**) Chromatograms presenting editing status for edits listed in the table (indicated by red arrows) that are observed in mock infected and infected cells after 24 and 48 hours post infection. Red rectangles indicate positions with observed A-I edits (recognized by the sequencing machine as A-G substitution). 24-, mock infected cells after 24h of culturing; 48-, mock infected cells after 48h of culturing; EDB-2_24 and EDB-2_48, cells infected with the EDB-2 strain after 24 and 48 hours, respectively; EDB-α-1_24 and EDB-α-1_48, cells infected with EDB-α-1 strain after 24 and 48 hours, respectively; Ref, reference nucleotide; Ed, edited nucleotide; In dbSNP, information if the edit is deposited in dbSNP.

Supplementary Table 1, related to figure 1. Variant table displaying nucleotide variants with a frequency greater or equal to 0.01 (1%). 10 RNAseq reads, obtained for analysed samples. Column names: Region: chromosome number; Position: location on the chromosome; Reference: nucleotide in reference sequence; Strand: strand information (1 for + strand, 0 for - strand and 2 for not-defined strand); Coverage-q30: depth per site at min. 30 quality score; MeanQ: mean quality score per site; BaseCount[A,C,G,T]: base distribution per site in the order A, C, G and T; AllSubs: the list of observed substitution at a given site separated by a space; Frequency: the frequency of an observed substitution, in case of multiple substitutions, it refers to the first reported in the AllSubs field; gCoverage-q30: depth per site at 30 min. quality score in WES/WGS data; gMeanQ: mean quality score per site in WES/WGS data; gBaseCount[A,C,G,T]: base distribution per site in the order A, C, G and T; gAllSubs: list of observed substitution at a given site separated by a space, '-' means invariant site; gFrequency: the frequency of an observed substitution, in the case of multiple substitutions, it refers to the first reported in the AllSubs field.

Supplementary Table 2, related to figure 4. Variant results after REDItools using stringent filtering scheme (sheets Ctrl24h, EDB-2-24h, EDB-a-1-24h, Ctrl48h, EDB-2-48h, EDB-a-1-48h) and statistics for substitution types (column "SubType") calculated by REDItools script (sheet StatsFromREDItools) in all locations (columns "ALL") and divided into sites located within Alu elements (column "ALU"), within repetitive elements other than Alu (column "REPnonALU") or

within non-repetitive locations (column "NONREP"). Column names: Region: chromosome number; Position: location on the chromosome; Reference: nucleotide in reference sequence; Strand: strand information (1 for + strand, 0 for - strand and 2 for not-defined strand); Coverage-q30: depth per site at min. 30 quality score; MeanQ: mean quality score per site; BaseCount[A,C,G,T]: base distribution per site in the order A, C, G and T; AllSubs: the list of observed substitutions at a given site separated by a space; Frequency: the frequency of the observed substitution, in case of multiple substitutions, it refers to the first reported in the AllSubs field; gCoverage-q30: depth per site at 30 min. quality score in WES/WGS data; gMeanQ: mean quality score per site in WES/WGS data; gBaseCount[A,C,G,T]: base distribution per site in the order A, C, G and T; gAllSubs: list of observed substitutions at a given site separated by a space, '-' means invariant site; gFrequency: the frequency of observed substitution, in case of multiple substitutions, it refers to the first reported in the AllSubs field; rmsk_feat: annotation with RepeatMasker (feature); rmsk_git: annotation with RepeatMasker (details); snp151_feat: annotation with dbSNP (feature); snp151_git: annotation with dbSNP (details); RefSeq_feat: annotation with RefSeq (feature); RefSeq_git: annotation with RefSeq (gene name); REDIportal[ed-knownEditing]: information if the substitution is deposed in REDIportal ("ed") or not ("-").

Supplementary Table 3. Gene expression data, related to figures 2 and 3. Data underlying figures 2 and 3 showing log2 transformed data to the left and Z-score data on the right of each table.

Supplementary Table 4, related to figures 4 and 5. Bioinformatics pipeline.
